# Non-casual Association Between Congenital Pulmonary Airway Malformations/Primary Lung Hypoplasia and Congenital Diaphragmatic Hernia (CDH)

**DOI:** 10.3389/fped.2020.00446

**Published:** 2020-08-04

**Authors:** Gloria Pelizzo, Sara Costanzo, Giorgio Giuseppe O. Selvaggio, Federico Rebosio, Lorena Canazza, Federica Marinoni, Valeria Calcaterra

**Affiliations:** ^1^Department of Biomedical and Clinical Science L Sacco, University of Milan, Milan, Italy; ^2^Department of Pediatric Surgery, Children's Hospital V. Buzzi, Milan, Italy; ^3^Pediatric and Adolescent Unit, Department of Internal Medicine, University of Pavia, Pavia, Italy; ^4^Pediatric Unit, Children's Hospital V. Buzzi, Milan, Italy

**Keywords:** congenital pulmonary airway malformations, primary lung hypoplasia, congenital diaphragmatic hernia, children, association

Congenital pulmonary airway malformations (CPAM), formerly congenital cystic adenomatoid malformations, represent the most common congenital developmental anomaly of the lung (1). This malformation is due to defects which can occur at different (embryological or fetal) stages during lung and tracheobronchial development (1). As reported by Leblanc et al. (2), CPAM classification is traditionally based on the proposition made by Stocker on the histological and morphological findings of lung lesions obtained via surgery. Stocker et al. (3) initially classified into three groups: type 1 with single or multiple large cysts of size (>2 cm) containing mucus cells; type 2 with multiple medium sized cysts (<2 cm), and possibly other anomalies associated; type 3 with bulky lesions with solid appearance and often mediastinal shift. Later, based on the site of lesion origin, this classification was expanded into five types by Stocker et al. (4), including also type 0 or acinar dysplasia, often lethal, and type 4 that is an acinar malformation lesion with varying sized cysts, all lined by type 1 and 2 alveolar cells with no mucous cells.

CPAM may be associated with other congenital anomalies, including congenital diaphragmatic hernia (CDH), which is characterized by a diaphragmatic defect, lung hypoplasia, and pulmonary hypertension. These anomalies are major determinants of survival in the neonatal period and predictors of long-term morbidity. Lung hypoplasia traditionally thought to be caused by impairment of lung growth by compression of visceral organs through CDH, eventhough, in animal models, pulmonary abnormalities have been described before the appearance of diaphragmatic herniation (5, 6).

In the literature, a few cases on the association between CPAM and CDH have been reported (7–10). Recently, a retrospective analysis of patients admitted to our Pediatric Surgery Unit for CPAM surgery, between July 2007 and January 2018, was conducted. We evaluated 86 cases (37 F/49 M) with a mean gestational age of 38 ± 2 weeks and a mean birth weight of 3,129.1 ± 540.4 g. In 68/86 of cases (79%) CPAM was prenatally diagnosed. In 3 of 86 CPAM patients (3.5%, 2 M/1 F), CDH was also detected. In detail (Table 1), patient 1 presented with a prenatal diagnosis of an hybrid lesion with left intra-lobar pulmonary sequestration (IPS) and CPAM (type 2 by Stocker); postnatally, a left CDH was associated with an esophageal duplication. In patient 2, we observed an IPS which overlapped with a CPAM (type 2 by Stocker); postnatally, a left CDH was detected. In patient 3, an radiological diagnosis of IPS associated with CPAM and right postnatal CDH were noted; only CDH repair was performed.

**Table 1:** Characteristics of the patients with the association between congenital pulmonary airway malformations (CPAM) and congenital diaphragmatic hernia (CDH).

	**Sex**	**Gestational age**	**Prenatal diagnosis**	**Type of CPAM**	**CDH**	**Other malformations**	**Outcome**
Case 1	M	Term	Yes (fetal MRI)	Intra-lobar pulmonary sequestration+CPAM 2^*^	Yes	Esophageal duplication	Good
Case 2	F	Term	No	Intra-lobar pulmonary sequestration+CPAM 2^*^	Yes	No	Good
Case 3	M	Preterm	No	Intra-lobar pulmonary sequestration+CPAM	Yes	No	Good

* *According to Stocker classification*.

This higher prevalence of CDH in our CPAM population, compared to the CDH prevalence of 2.4–4.9 per 10,000 births reported in the literature (11, 12), supports the hypothesis of a causal association between CPAM and CDH. Lung damage may be an isolated event or may be associated with diaphragm defect leading to CDH (5, 6).

Several theories regarding development of the diaphragm have been proposed as the cause of CDH. One of these suggests that abnormal lung development also leads to abnormal development of the diaphragm allowing herniation of abdominal contents into the thoracic cage. As reported by Iritani, when lung bud development is disturbed, there is impaired development of the post hepatic mesenchymal plate (mesenchymal tissue of dorsal liver), which is closely related to lung growth; the result is a defective diaphragm (6).

In humans, lung development begins at 3–4 weeks' gestation and includes six different morphological stages that correspond with key developmental transitions: embryonic (3–7 weeks' gestation), pseudoglandular (6–17 weeks' gestation), canalicular (16–26-weeks' gestation), saccular (24–38 weeks' gestation), alveolar (36 weeks' gestation to 2 years of age) and microvascular maturation (birth to 2–3 years of age) (1, 13). A multitude of factors have been identified that direct the highly predetermined lung program, these include genes, such as GATA4, FOXA2, SOX2, FGF1–2, transcription factors, growth factors and their receptors, extracellular matrix proteins, and intercellular adhesion molecules (1, 14), The timing of an embryologic insult generally correlates with the type of CPAM lesion and histopathologic findings.

As observed in CPAM, Dalmer and Clugston (1), reported an increase in the number of candidate disease-causing genes, these have been associated with CDH and are significantly enriched in relation to different CDH clinical presentations. Some of these genes are also involved in lung embryogenesis, i.e., RAR, GATA4, and FOXA2, FGFR2, IGF2, SOX2 (1). Similarly, perturbed growth factor signaling, for example fibroblast and endothelial growth factors, may affect different embryonic phases, directing abnormal signals to the developing diaphragm and lung.

On the other hand, the coexistence of common pathogenic mechanisms may support the theory of a non-casual association between CPAM and CDH. Lung development begins at 4 weeks' gestation and normal diaphragmatic closure occurs substantially later at 8 weeks (5). This chronology suggests that the initial insult occuring during organogenesis could first involve the lung. CPAM associated with CDH points to an early defect in embryogenesis, prior to the 6th week of gestation, in most of the cases in the early pseudoglandular stage of development, before complete fusion of the diaphragm (5). Therefore, the phenotype of the associated malformation may be related to the timing of the insult. If we accept this hypothesis, lung hypoplasia could be the primary causal factor in the pathophysiology of CDH (5, 6). In fact, as reported by Iritani, (6) if the development of lung bud is disturbed, an impaired development of a post-hepatic mesenchymal plate (closely related to the lung development) occurs, resulting in a defective diaphragm.

Studies in developmental biology (14) and future translational research should ameliorate our knowledge on the pathogenesis of these congenital anomalies and help define whether the presence of both malformations in a newborn is a sign that they had a significant role in the global embryopathy.

## Author Contributions

GP and VC made a substantial contribution to the concept or design of the work, drafted the article, and revised it critically for important intellectual content. SC, GS, FR, LC, and FM made a substantial contribution to the concept or design of the work and drafted the article. All authors approved the version to be published.

## Conflict of Interest

The authors declare that the research was conducted in the absence of any commercial or financial relationships that could be construed as a potential conflict of interest.
